# Assessing the Reliability of Postoperative Cross-Table Radiographs Compared to CT Scans for Measuring Acetabular Anteversion Angle Following Total Hip Arthroplasty

**DOI:** 10.7759/cureus.68575

**Published:** 2024-09-03

**Authors:** Adhithya Kaushik, Vinay Kumar C, Anoop Hegde, Lawrence Mathias, Vikram Shetty

**Affiliations:** 1 Orthopaedics, Meenakshi Medical College Hospital and Research Institute, Kanchipuram, IND; 2 Orthopaedics, K S Hegde Medical Academy, Mangalore, IND

**Keywords:** computed tomography, acetabulum, hip prosthesis, radiography, hip arthroplasty

## Abstract

Aim: This study aimed to evaluate acetabular anteversion following total hip arthroplasty (THA) using cross-table lateral radiographs and CT scans and to determine the reliability of cross-table lateral radiographs compared to computed tomography (CT) scans for measuring acetabular anteversion.

Materials and methods: We conducted a cross-sectional study of patients undergoing THA at Justice K.S. Hegde Charitable Hospital, Deralakatte, Mangalore, from January 2020 to June 2021. Radiographs are typically taken in both anteroposterior (AP) and lateral views after THA. However, for this study, a cross-table lateral view was used instead of the usual lateral radiograph to measure the angle of anteversion. The anteversion was calculated using the method described by Woo and Morrey. Additionally, CT scans were performed on all patients as part of the study protocol. The anteversion measured in these scans was compared to that in the cross-table radiographs to assess the latter’s reliability for routine use. The risk of radiation exposure from CT scans was minimized by adhering to the ALARA (As Low as Reasonably Achievable) principle, with only axial sections of the acetabular cup being scanned.

Results: The results show that the radiographic acetabular anteversion and CT scan measurements have a mean difference of 0.3036. There is a positive correlation between these measurements. The p-value is not statistically significant (p=0.698). Therefore, the measurements are correlated with each other with a linear relationship (r=0.919). For anteversion measurements using the X-ray method, the mean was 27.16 degrees with a standard deviation of ±9.49. The median was 27.26 degrees, ranging from 10.27 to 42.58 degrees. In comparison, the CT method yielded a mean anteversion of 27.40 degrees with a standard deviation of ±8.50 degrees. The median was 27.64 degrees, ranging from 12.35 to 43.11 degrees.

Conclusion: Cross-table lateral radiographs are a reliable and comparable method to CT scans for measuring acetabular anteversion following total hip arthroplasty.

## Introduction

The success and stability of total hip arthroplasty (THA) depend on several factors, with the positioning of the acetabular component being one of the most crucial. Incorrect placement of the acetabular cup can lead to complications such as implant wear, reduced range of motion, and dislocation. While CT scans are considered the gold standard for measuring acetabular anteversion, their high cost, limited availability, and radiation exposure make them less practical for routine use.

Understanding the biomechanics of the hip cup after THA is essential, as misalignment can have serious consequences. Acetabular anteversion refers to the angle between the cup axis and the patient’s coronal axis [1]. Maintaining specific anteversion values is essential for ensuring a stable hip joint. Historically, Lewinnek’s “safe zones” suggested an ideal anteversion angle of 15 degrees, with a tolerance of ±10 degrees [2]. However, subsequent research has shown that dislocations can occur within these safe limits. These dislocations are often due to a combination of factors, including patient-specific characteristics, disease conditions, spino-pelvic interactions, soft tissue issues, and prosthesis-related factors [3]. Consequently, strict adherence to Lewinnek’s safe zones is being reassessed, with ongoing research aiming to define more accurate safety margins.

Replicating the native anatomy of the hip with artificial prosthetics remains challenging. Excessive anteversion increases the risk of anterior dislocation, while insufficient anteversion may lead to implant wear and impingement, significantly if the abduction angle deviates from normal limits. Studies have linked increased wear and osteolysis to abduction angles greater than 45 degrees [4]. Key factors influencing anteversion and abduction include the surgical approach and body mass index (BMI) [5]. Intraoperative guides, such as external mechanical tools or internal markers like the transverse acetabular ligament, assist in measuring anteversion, but cup inclination must also be considered [6].

Currently, a patient-specific approach to acetabular anteversion is gaining attention. This method accounts for individual anatomical and other factors, providing a more tailored solution for each patient. Although this approach is gaining traction in joint replacement centers worldwide, further research is needed to establish it as a standard practice. The debate continues over the applicability of Lewinnek’s safe zones, as these may not fully address all patient-specific factors [6].

Various radiographic methods have been explored for measuring anteversion. Conventional lateral radiographs do not provide a clear view of the acetabular cup, making anteversion measurement difficult [3,4]. Anteroposterior (AP) radiographs can measure anteversion but require complex algorithms and software, which can be impractical. Cross-table radiographs, which involve positioning the patient supine, are more accurate and comfortable for assessing anteversion [3]. While CT scans are the most accurate method for measuring acetabular anteversion, their cost and radiation risks limit their routine use [6]. This study aimed to compare the reliability and accuracy of cross-table radiographs with CT scans for evaluating cup anteversion.

## Materials and methods

The study was conducted on patients undergoing total hip arthroplasty (THA) at Justice K S Hegde Charitable Hospital, affiliated with K S Hegde Medical Academy, a constituent of NITTE (Deemed to be University), located in Deralakatte, Mangalore. This cross-sectional, hospital-based study spanned from January 2020 to June 2021. The sample size was determined using G STAR POWER software (Heinrich-Heine-Universität Düsseldorf, Düsseldorf, Germany), considering a 5% level of significance, 80% power, and an effect size of 0.25 for three different techniques, resulting in a required sample size of 25. Convenient sampling was employed.

The inclusion criteria consisted of patients undergoing THA at the hospital above, while those unable to flex the contralateral hip to 90 degrees were excluded from the study. Ethical clearance was obtained from the Institutional Review Board of K S Hegde Medical Academy, Nithyananda Nagar, before the commencement of the study (approval number: INST.EC/EC/097/2019-20), and informed consent was obtained from all participants.

**Figure 1:**
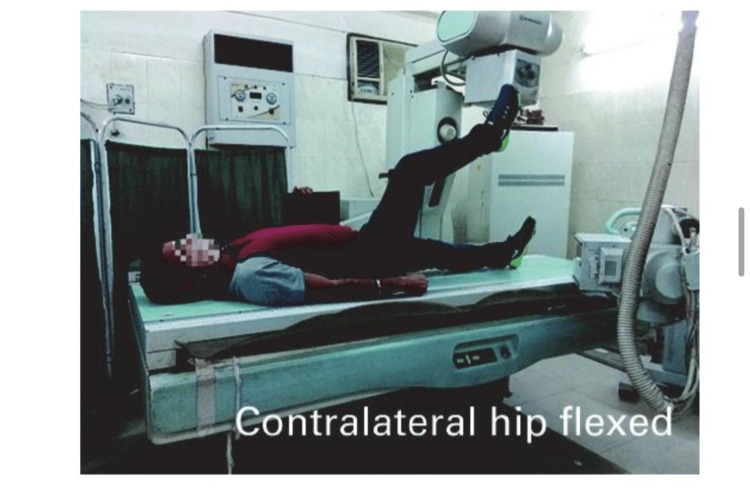
Position of patients

The patient was positioned supine with the contralateral hip flexed, the operative hip internally rotated by 15 degrees, and the X-ray beam parallel to the floor. Fluoroscopy was used to guide the positioning and ensure accurate imaging. The 15 degrees internal rotation was critical for obtaining a clear view of the femoral head and acetabulum, which was essential for assessing the alignment and positioning of the prosthetic components in total hip arthroplasty (Figure 1).

Radiographs typically take in anteroposterior (AP) and lateral views after THA. However, for this study, a cross-table lateral view was used instead of the usual lateral radiograph to measure the anteversion angle. The anteversion was calculated using the method described by Woo and Morrey. Additionally, CT scans were performed on all patients as part of the study protocol. The anteversion measured in these scans was compared to the cross-table radiographs to assess the latter’s reliability for routine use. The risk of radiation exposure from CT scans was minimized by adhering to the As Low as Reasonably Achievable (ALARA) principle, with only axial sections of the acetabular cup being scanned.

Data collection involved recording demographic details, clinical history, and radiographic measurements. The data was anonymized to maintain patient confidentiality. Two independent observers performed all measurements to ensure interobserver reliability. Discrepancies were resolved through consensus.

Statistical analysis was conducted using repeated measures ANOVA, with a significance level set at p<0.05. Descriptive statistics were used to summarize demographic and clinical characteristics. Interobserver reliability was assessed using the intraclass correlation coefficient (ICC). Bland-Altman plots were utilized to visualize the agreement between radiographic and CT anteversion measurements.

Quality assurance measures included periodic calibration of radiographic equipment and training sessions for radiographers to standardize the imaging protocol. A steering committee monitored the study to ensure adherence to the protocol and address any issues arising during the study period.

## Results

The study included participants of various ages. The largest group was between 51 and 60 years old, with 12 participants (48%), a difference that was statistically significant (p = 0.001). The gender distribution comprised 15 males (60%) and 10 females (40%), with no significant difference (p = 0.31). Most surgeries were performed on the right side, with 17 cases (68%), compared to the left side with eight cases (32%), although this difference was not statistically significant (p = 0.07). Regarding the type of total hip arthroplasty, uncemented procedures were most common, with 17 cases (68%), followed by cemented procedures with seven cases (28%), and hybrid procedures with one case (4%). This distribution was statistically significant (p = 0.0003) (Table 1).

**Table 1:** Demographic characteristics, surgery site, and type of both groups p< 0.05 is considered significant. * indicates significant p-value.

Variables	Total number of cases N (%)	Chi-square test	p-value
Age (in years)		18.92	0.001^*^
< 30	2 (8%)		
31 to 40	3 (12%)		
41 to 50	3 (12%)		
51 to 60	12 (48%)		
61 to 70	4 (16%)		
> 70	1 (4%)		
Sex		1	0.31
Male	15 (60%)		
Female	10 (40%)		
Surgery side		3.24	0.07
Left	8 (32%)		
Right	17 (68%)		
Type of total hip arthroplasty		15.68	0.0003^*^
Cemented	7 (28%)		
Hybrid	1 (4%)		
Uncemented	17 (68%)		

The study compared anteversion measurements obtained using X-ray and CT methods both before and after surgery. Before surgery, the average anteversion was 27.17 ± 9.49 degrees with X-ray and 27.47 ± 8.56 degrees with CT, showing no significant difference (t-test value = 0.40, p = 0.69). After surgery, the measurements were 27.16 ± 9.49 degrees with X-ray and 27.4 ± 8.5 degrees with CT. This comparison also indicated no significant difference (t-test value = -0.103, p = 0.918) (Table 2).

**Table 2:** Comparison of anteversion before and after surgery p< 0.05 is considered significant.

Variables	X-ray method	CT method	t-test value	p-value
Anteversion measurements before surgery	27.17 ± 9.49	27.47 ± 8.56	0.40	0.69
Anteversion measurements after surgery	27.16 ± 9.49	27.4 ± 8.5	-0.103	0.918

**Figure 2:**
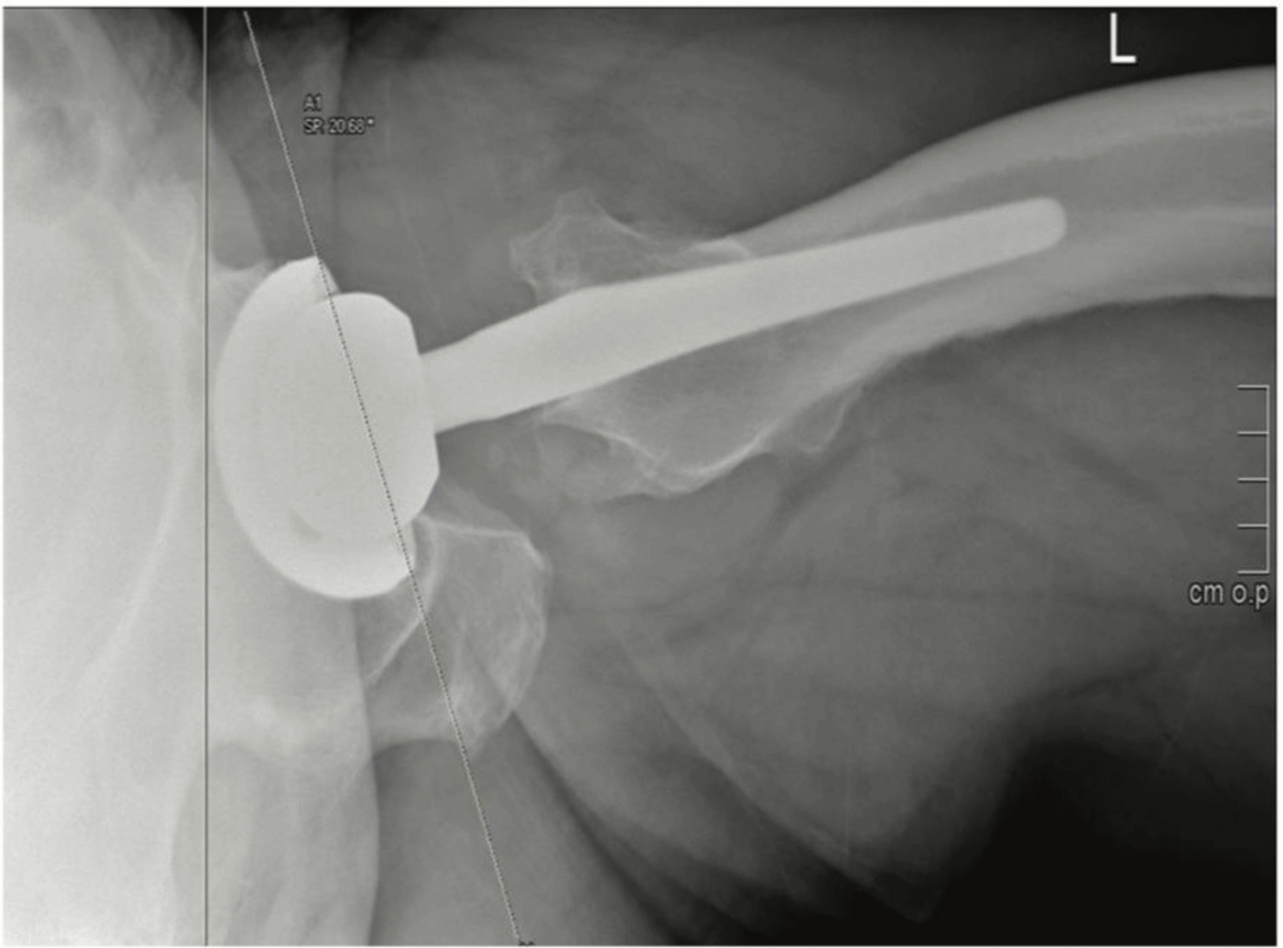
The Woo and Morrey method represents the version as the angle between a perpendicular line and a tangential line to the acetabular component's opening face

**Figure 3:**
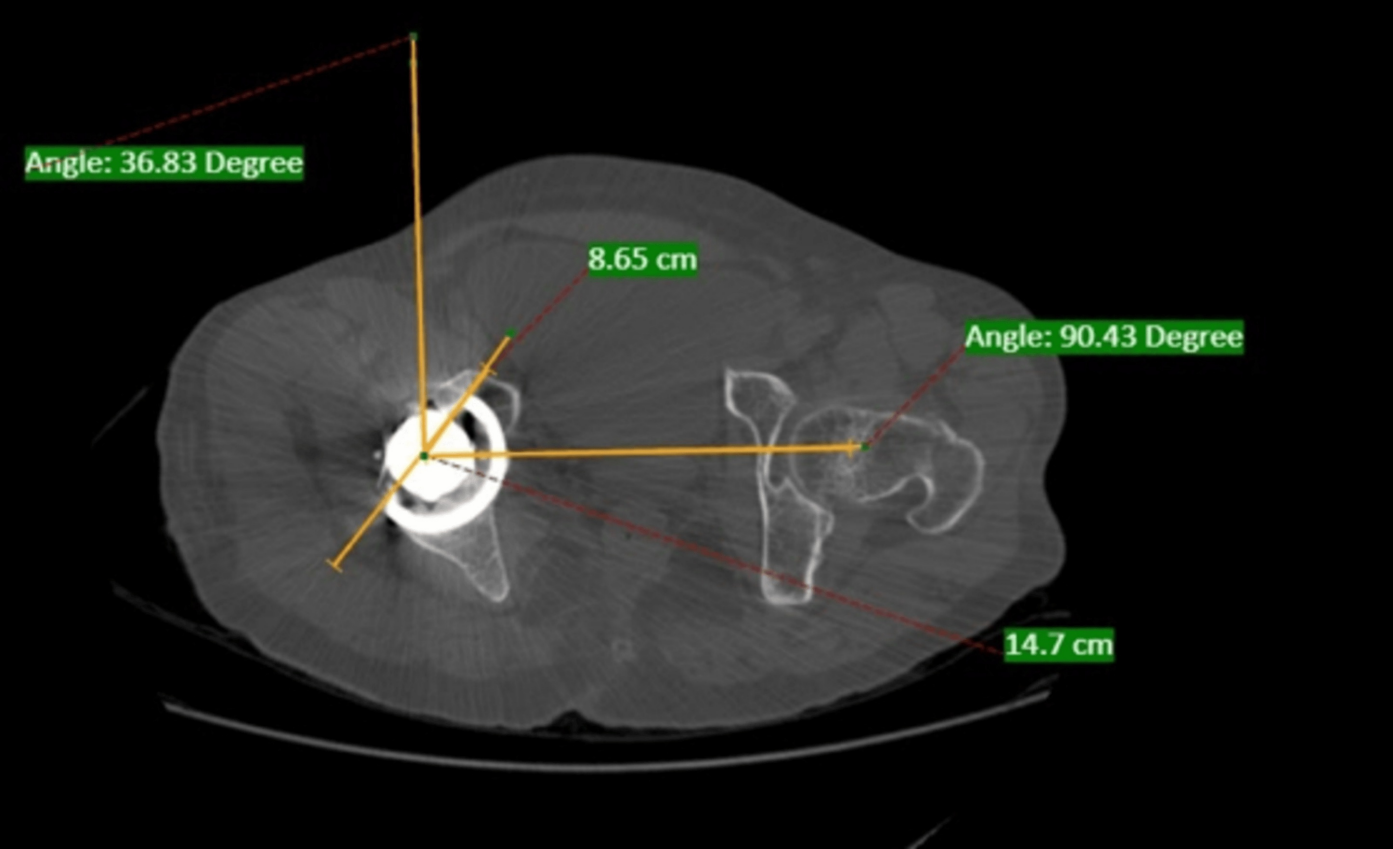
CT scan done in our study

Figure 3 demonstrated the Woo and Morrey method for measuring the version of the acetabular component in total hip arthroplasty. In this method, a perpendicular line was drawn vertically on the radiograph, and a tangential line was aligned with the opening face of the acetabular component. The angle formed between these two lines, referred to as the version angle, was then calculated. This technique was essential for assessing the orientation of the acetabular component, ensuring accurate positioning to minimize the risk of complications such as dislocation and to optimize the long-term outcomes of the hip replacement (Figure 2).

**Figure 4:**
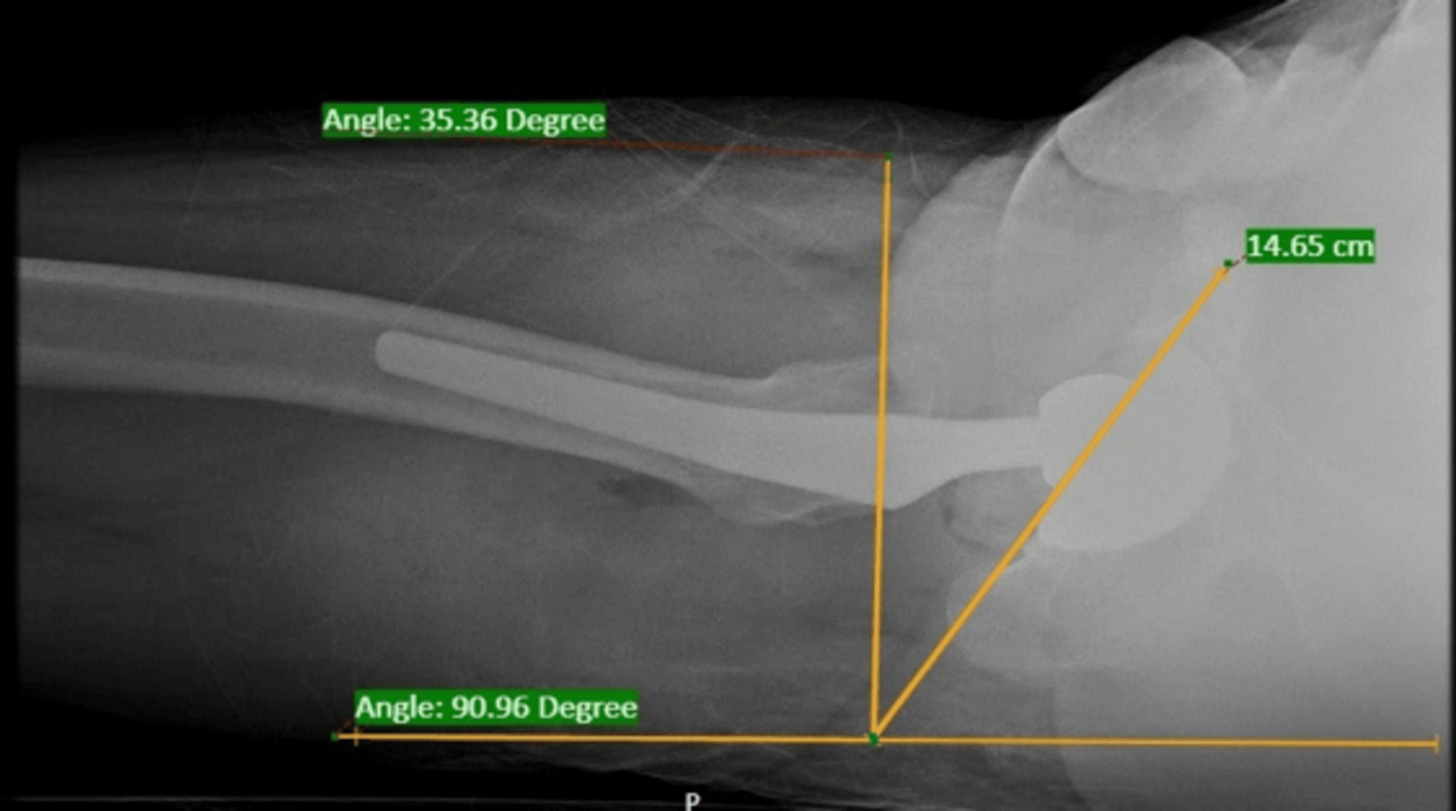
Cross-table radiograph done in our study

The results show that the radiographic acetabular anteversion and CT scan measurements have a mean difference of 0.3036. There is a positive correlation between these measurements. The p-value is not statistically significant (p = 0.698). Therefore, the measurements are correlated with each other with a linear relationship (r = 0.919). The final results indicate that both CT and radiographic measurements are correlated in assessing acetabular anteversion after THA. Figure 3 shows anteversion in CT imaging, while Figure 4 presents X-ray findings.

## Discussion

In the literature, acetabular anteversion is most commonly measured using anteroposterior (AP) radiographs. Markel et al. [7] described a method where separate AP views of the pelvis and the operated side are compared to calculate anteversion. However, cross-table lateral radiographs are superior for measuring anteversion following total hip arthroplasty (THA). Goyal et al. [8] introduced a software technique for measuring anteversion from AP radiographs by connecting bony landmarks with imaginary lines. This method faced challenges in drawing precise lines, leading to variability among observers and questioning its reliability.

Digital software often uses two methods to measure anteversion on AP radiographs: those developed by Liaw et al. and Widmer [9,10]. These methods calculate anteversion by measuring the anteroposterior and transverse diameters of the acetabular cup and their ratio. Studies show that the mean acetabular anteversion on cross-table radiographs was 53.1 ± 10.7 degrees (ischiolateral method) [9,10]. For AP radiographs, Widmer’s method yielded a mean anteversion of 21.4 ± 3.6 degrees, while Liaw’s method yielded 20.3 ± 4.8 degrees. The mean anteversion measured by CT scans was 26.02 ± 6.8 degrees [11]. There was a strong correlation between CT measurements and the Liaw [9] (p < 0.001) and Widmer [10] (p < 0.001) methods for AP radiographs [12].

Other methods for measuring anteversion on AP radiographs, described by Pradhan [13], Lewinnek et al. [14], and Hassan et al. [15], also calculate anteversion by measuring ellipsoid diameters and their ratios. Liaw’s method showed the highest accuracy, with a mean difference of 1.37 ± 1.73 degrees from reference angles [12]. Since anteversion is a three-dimensional concept, measuring it from biplanar radiographs can be oversimplified, prompting ongoing research for more optimal methods [16].

In our study, we compared cross-table radiographs with CT scans for measuring acetabular anteversion. Cross-table radiographs offer benefits in patient positioning and are less cumbersome postoperatively. Literature supports using both AP and lateral radiographs, but cross-table radiographs are often preferred due to their advantages. Traditional safe zones for anteversion, as described by Lewinnek, are increasingly seen as inadequate in favor of more individualized approaches that consider each patient’s unique pelvic anatomy and soft tissue status.

Our study, involving 25 patients, found the mean anteversion to be 27.16 degrees on radiographs and 27.47 degrees on CT scans. Lu et al. [17] observed similar mean anteversion values, 17.9 degrees for radiographs and 17.4 degrees for CT scans, indicating no significant difference. Hernández-Vicente et al. [18] found a mean anteversion of 13.9 degrees on radiographs and 17.8 degrees on CT scans, while Oh et al. [19] reported 14.3 to 14.5 degrees on radiographs and significantly lower values on CT scans, suggesting that radiographs may be less precise.

Our study utilized cross-table lateral radiographs, where the patient is supine, with the beam directed from the opposite side, the contralateral hip flexed to 90 degrees, and the ipsilateral limb rotated 10 to 15 degrees internally. We found mean anteversion values of 27.16 degrees on radiographs and 27.47 degrees on CT scans. Pankaj et al. [1] found cross-table radiographs comparable to CT scans, with CT scans as the gold standard. Their study showed mean anteversion values of 18.35 degrees (Woo and Morrey method), 51.45 degrees (ischiolateral method), and 21.22 degrees (CT scans), with no statistically significant differences between radiographs and CT scans. Raj et al. [20] also found Woo and Morrey’s methods comparable to CT scans, with differences not statistically significant (p = 0.272). However, the ischiolateral method showed significant differences (p < 0.001).

Various factors contribute to inaccuracies in AP radiographs, including pelvic rotation. Correction methods such as Einzel-Bild-Röntgen-Analyse (EBRA), Radiostereometric Analysis (RSA), and 2D/3D registration techniques attempt to address these issues, but lateral radiographs are more effective for measuring pelvic tilt [21]. Saka et al. [22] described a method where legs hang off the radiographic table to correct pelvic rotation, showing promising results with no significant difference from CT measurements (p = 0.207). While AP radiographs can be helpful, lateral radiographs and advanced digital software offer better accuracy and reliability.

Given these factors, cross-table lateral radiographs are the most accurate, reliable, and least error-prone method for measuring acetabular anteversion following THA. Nomura et al. [23] found that Widmer’s method had the highest correlation with CT scans (p = 0.088) and a mean difference of -0.9 degrees. This study concluded that Widmer’s method is the most accurate for AP radiographs. However, since pelvic rotations and tilts vary by patient, the most reliable measurements come from methods independent of such factors. Cross-table lateral radiographs offer consistent results and minimal observer error, making them highly recommended for assessing acetabular anteversion.

Limitations of the study

This study has several limitations. Firstly, the cross-sectional design prevents the establishment of causality and the assessment of temporal changes. The sample size of 25 patients, selected through convenience sampling, may not adequately represent the broader population undergoing total hip arthroplasty (THA). Additionally, excluding patients who could not flex the opposite hip to 90 degrees restricts the applicability of the findings.

The single-center setting of the study limits the generalizability of the results to other healthcare environments. The absence of long-term follow-up data restricts the evaluation of the durability and extended outcomes of the procedures under investigation. Additionally, the lack of detailed intraoperative data limits our understanding of surgical nuances that might affect outcomes, and the relatively short postoperative follow-up period hinders the assessment of long-term complications and prosthesis performance. Future research should include larger sample sizes, extended study periods, and multicenter participation to validate these findings and enhance their generalizability.

## Conclusions

In conclusion, cross-table radiographs have proven to be reliable and comparable to CT scans for measuring acetabular anteversion following total hip arthroplasty. Our study found that the mean anteversion measurements obtained from both cross-table radiographs and CT scans were statistically similar, indicating that cross-table radiographs can effectively serve as an alternative to CT scans in clinical practice. This finding supports the use of cross-table radiographs due to their practical advantages, such as ease of patient positioning and reduced postoperative inconvenience, while still providing accurate anteversion measurements comparable to those obtained from CT scans.
